# Mapping the Alterations of Glutamate Using Glu-Weighted CEST MRI in a Rat Model of Fatigue

**DOI:** 10.3389/fneur.2020.589128

**Published:** 2020-10-29

**Authors:** Ruili Li, Zhuozhi Dai, Di Hu, Haiyan Zeng, Zeman Fang, Zerui Zhuang, Haiyun Xu, Qingjun Huang, Yilong Cui, Handi Zhang

**Affiliations:** ^1^Department of Psychiatry, Mental Health Center of Shantou University, Shantou, China; ^2^Department of Radiology, Shantou Central Hospital, Shantou, China; ^3^Department of Radiology, The Second Affiliated Hospital of Shantou University Medical College, Shantou, China; ^4^Laboratory for Biofunction Dynamic Imaging, RIkagaku KENkyusho/Institute of Physical and Chemical Research (RIKEN) Center for Systems Dynamics Research, Kobe, Japan; ^5^Mental Health Center, Xianyue Hospital, Xiamen, China; ^6^School of Psychiatry, Wenzhou Medical University, Wenzhou, China

**Keywords:** glutamate, fatigue, chronic fatigue syndrome, spontaneous activity, glutamate weighted chemical exchange saturation transfer (Glu-weighted CEST)

## Abstract

**Objective:** Glutamate dysregulation may play an important role in the pathophysiology of fatigue. Glutamate weighted chemical exchange saturation transfer (Glu-weighted CEST) MRI is a recently developed technology which enables measuring glutamate *in vivo* with high sensitivity and spatial resolution. The purpose of this study is to map the alternations of brain glutamate in a rat model of fatigue.

**Methods:** Rats were subjected to 10 days fatigue loading procedure (fatigue group) or reared without any fatigue loading (control group). Spontaneous activities of rats in the fatigue group were recorded from 3 days before fatigue loading to 4 days after the end of fatigue loading. Glu-weighted CEST were performed following 10-day fatigue loading.

**Results:** Rats in the fatigue group exhibited significant reduced spontaneous activities after 10-day fatigue loading. The glutamate level in the whole brain increased significantly in the fatigue group compared to that in the control group. Further analysis of glutamate in the sub-regions of brain including the prefrontal cortex, hippocampus, and striatum revealed a trend of increment, although statistical significance was not reached.

**Significance:** The increase of glutamate level in the brain may be a crucial process involved in the pathophysiology of fatigue.

## Introduction

Fatigue is one of the most common symptoms reported in many psychiatric and neurological disorders, including major depressive disorder (MDD), anxiety disorder, chronic fatigue syndrome (CFS), multiple sclerosis (MS), Parkinson's disease, traumatic brain injury, myasthenia gravis, stroke, and amyotrophic lateral sclerosis (1, 2). Although many efforts have been put into this field, the current treatments are not yet satisfactory, and the underlying molecular and cellular mechanisms related to fatigue remain unclear.

Glutamate is the most abundant free amino acid and the major excitatory neurotransmitter in the mammalian central nervous system (CNS). It plays important roles in varied brain possesses including protein synthesis, nitrogen homeostasis, energy metabolism, and synapse transmission. Glutamate signaling is crucial in information intake and processing within the CNS. Impaired glutamate neurotransmission has been proposed as one of the potential pathophysiological mechanisms of mental fatigue, which appears to be a consequence of a compromised ability to intake and process information (3).

There are accumulating evidences indicating the involvement of glutamate dysregulation in the pathophysiology of fatigue. Previous studies focusing on exercise-induced fatigue demonstrated that a short-term vigorous exercise lead to an increase of cortical glutamate level as measured by proton magnetic resonance spectroscopy (^1^H-MRS) in healthy human volunteers (4, 5). In addition, rats exhibited increased glutamate and glutamine ^1^H-MRS signals in the brain following an acute exhaustive exercise (6), and strenuous exercise induced an increase of extracellular glutamate concentration and increased expression of glutamate transporter-1 (GLT-1), a member of excitatory amino acid transporters accounting for glutamate transportation, which might mediate the exercise-induced glutamate changes in CNS (7). Glutamate signaling may also play an important role in the development of fatigue in some chronic conditions, such as MS, CFS, and cancer-related fatigue (8–10). Anti-glutamate medicines, including memantine, and riluzole, were found to have effect of worsening fatigue on MS patients (11, 12). A ^1^H-MRS study found that hypothalamus glutamate levels were correlated with fatigue scores in MS patients (13). By utilizing whole genome microarray data, a previous genetic study identified two glutamate receptor genes, glutamate receptor ionotropic kinase 2 (*GRIK2*), and glutamate ionotropic N-methyl-D-aspartate 2B (*GRIN2B*), were associated with CFS (8). Moreover, the decreased expression of *GRIK2* and glutamate receptor metabotropic 4 (*GRM4*) and the increased expression of *GRM1* were found in CFS subjects compared to non-fatigue controls in this study, implicating glutamate signaling dysfunction in the process of chronic fatigue (8). Interestingly, glutamate receptor signaling was also identified as a key pathway mediating post-radiotherapy fatigue development in cancer patients (10).

However, not all the studies found an association between glutamate levels and fatigue. Several metabolomics analyses on the blood and urine samples of CFS patients revealed no alterations of glutamate levels compared to healthy controls (14–16). A previous ^1^H-MRS study investigating cortical amino acid neurotransmitter function of CFS patients also did not find any difference in the combined glutamate and glutamine signals compared to healthy subjects or MDD patients (17). One animal study showed that acute excise induced a slight decrease of glutamate levels in rats administrated with prolonged exercise (18).

The aforementioned mixed results may reflect different glutamate-mediated responses to fatigue under the different context. In spite of the progress, the relationship between glutamate and fatigue, especially chronic fatigue, remains not fully understood. Although ^1^H-MRS is widely employed to measure the levels of glutamate and other metabolites in the brain, there are some limitations such as low spatial resolution and longer acquisition time. Glutamate weighted chemical exchange saturation transfer (Glu-weighted CEST) magnetic resonance imaging (MRI), a recently developed method, enables indirect measurement of glutamate *in vivo* by probing the proton exchange of glutamate amine with bulk water, therefore providing higher sensitivity, and spatial resolution than conventional ^1^H-MRS (19). Glu-weighted CEST imaging has previously been demonstrated successful in animal models (20–22) and in schizophrenia patients (23).

In the present study, we investigated the glutamate levels with Glu-weighted CEST technology in an established animal model of prolonged fatigue (24, 25). We measured the spontaneous activity of animals in their own homecages and glutamate levels in the whole brain and brain regions relevant to the pathophysiology of fatigue following 10-day chronic fatigue loading.

## Materials and Methods

### Animals

Five-week old Sprague-Dawley (SD) rats weighting around 200–230 g were purchased from Vital River Laboratories (Beijing, China). Two batch of rats were used for the study. The first batch of rats (*n* = 5) were used for spontaneous activity measurement. The secondary batch of rats (*n* = 16) were used for multi-parametric MRI experiment. The animals were acclimated for 1 week before experiments in controlled environment (23° ± 1°C with lights on at 7:00 and off at 19:00), with *ad libitum* access to a standard laboratory diet and tap water. Rats were handled with gentle touch twice every day so that they were familiar with the experimenter. All of the experimental procedures were approved by the Ethics Committee of Shantou University Medical College on animal care and use.

### Fatigue Model and Experimental Procedures

**Figure 1:**
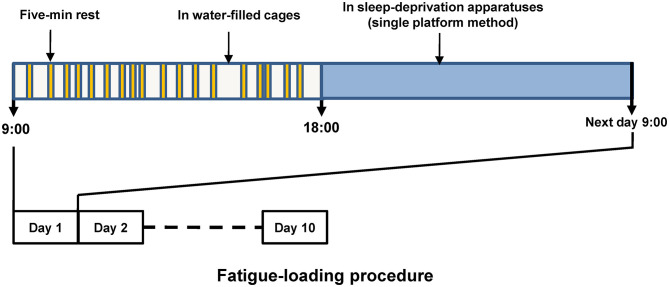
Schematic diagram of fatigue loading procedure. The upper part shows the routine procedure performed in each of the fatigue-loading days, and the procedure was repeated over this 10-day fatigue loading period. Five-min rest was given twice per hour, but the timing within each hour was arranged randomly to prevent the adaptation generated by fixed intervals.

We established the fatigue model with rats based on the previous reports with some modifications (24, 25). The procedure of fatigue loading was shown in Figure 1. Briefly, after acclimation for 1 week, the rats were randomly assigned to either a control or a fatigue group. Rats in the fatigue group were exposed to continuous fatigue loading for 10 days. During the daytime (from 9:00 to 18:00), the rats in the fatigue group were singly kept in cages filled with water (23°C ± 1°C) to a height of 2.2 cm and were allowed to rest for 5 min twice per hour throughout the 10 days of fatigue loading. During the 5-min rest, the rats were transferred to a standard cage and then returned to the previous water-filled cage. The water was changed once per day. During the nighttime (from 18:00 to 9:00), the rats were transferred to a sleep-deprivation apparatus (single platform method) to deprive their rapid eye movement (REM) sleep for the whole night as described before (26). The sleep-deprivation apparatus consisted of a single round platform (diameter: 6.3 cm) in an enclosed tank, and the platform was surrounded by water to about 1 cm below platform surface. The water in the tank was changed once per day. The rats in the control group were reared singly in standard cages without any fatigue loading except handled by the experimenter every day.

### Measurement of Spontaneous Activity

The 24-h spontaneous activity of each rat was recorded with Nano tag® (Kissei Comtec Co, Nagano, Japan) for evaluation of fatigue state. The Nano tag® was implanted subcutaneously between the scapulae of each rat according to the manufacture's instruction. Briefly, the implanting operation was performed 10 days before fatigue-loading procedure under anesthesia induced by 2% pentobarbital sodium. After the operation, 100 mg/kg ampicillin sodium was injected subcutaneously for each rat to prevent infection. The rats were allowed to recover for 10 days before the fatigue loading procedure. All the rats were individually housed in their home cages. After 10 days of recovery from the operations, these rats were as active as the normal rats. The level of spontaneous activity was recorded from 3 days before fatigue loading to 4 days after the end of the fatigue-loading procedure. The spontaneous activity level was normalized to the mean value of the 3 days before fatigue loading. Spontaneous activity was analyzed using the Nano-tag viewer program (Kissei Comtec Co., Nagano, Japan).

### Multi-Parametric MRI Acquisition

A second batch of rats were used for multi-parametric MRI, including glutamate imaging, conventional T2 weighted imaging, T1 map and T2 map, following the end of fatigue loading. MRI was performed on a 7T animal scanner (Agilent Technologies, Santa Clara, CA) with a 63 mm internal diameter standard ^1^H volume coil for RF transmission and reception. Field gradients: 400 mT/m in maximum 200A. MRI was performed under 1–2% isoflurane anesthesia and 1 L/min oxygen administration. The respiration rate and body temperature of rats were monitored using a MRI compatible monitoring system (Small Animal Instruments, Inc., USA). Rectal temperature was maintained at 37°C using a warm air blown inside the bore of the magnet.

Before MRI acquisition, the B0 field was first corrected by 3D gradient shimming, which adjusted high-order gradient shimming currents according to the derived B0 map. Then, the RF field and center frequency were calibrated in the prescan protocol. Glu-weighted CEST imaging was obtained using a continuous wave RF spoiled gradient echo sequence in the coronal plane, with a saturation power of 5.9 μT (250 Hz) and a saturation time of 2 s. The saturation frequency ranged from 5 ppm to −5 ppm with an interval of 0.2 ppm. Control images (I0) were obtained at an offset frequency of 100 ppm for normalization, and Glu-weighted CEST was measured at 3 ppm. Other sequence parameters were as follows: FOV = 50 × 50 mm.TR = 17.58 ms; TE = 2.77 ms, flip angle = 15 deg, number of average = 8, matrix size = 128 × 64, slice thickness = 2 mm, bandwidth = 50 kHz, k-space sampling = linear. T2 weighted imaging was acquired using 2D rapid acquisition with a fast spin echo sequence in the same plane. The T2 weighted imaging parameters were as follows: TE/TR = 40/3,000 ms, repetitio*n* = 1, slice thickness = 2 mm, imaging matrix size = 256 × 256; number of averages = 2.

B0 map, B1 map, T1 map, and T2 map were obtained from the same slice, as described in our previous studies (27–29). Specifically, the B0 map was obtained by dual echo method with delta TE of 2 ms. B1 map was obtained using the double-angle method with two flip angles of 60 deg and 120 deg. T1 map was obtained using inversion recovery sequence, with seven inversion times (TI) ranging from 100 to 7,500 ms. T2 map used the spin-echo echo-planar imaging sequence, with five echo times ranging from 40 to 400 ms.

All data was processed in MATLAB 2017a (Mathworks, Natick MA, USA). The T1 and T2 map were generated from least-squares mono-exponential fitting of signal intensities. The Z-spectral was plotted using normalized signal intensity (I/I0) as a function of saturation frequency. The zero frequency of Z spectral was realigned and corrected with B0 map voxel-by-voxel. The Glu-weighted CEST image was calculated by the following equation: Glu-weighted CEST = (I(-3 ppm)—I(+3 ppm))/I0, in which I(-3 ppm) and I(+3ppm) were images with frequency offset of −3 ppm and +3 ppm, respectively.

### Statistical Analysis

All data were expressed as mean ± standard error of mean (SEM). One-way analysis of variance (ANOVA) was applied to compare spontaneous activity of rats in fatigue group at different time points. *Post-hoc* pairwise comparisons were performed with Bonferroni method. The two-tailed Student's *t-*test was performed to compare levels of glutamate between both groups in the whole brain or varied brain regions. Differences were considered statistically significant at *P* < 0.05.

## Results

### Decrease of Spontaneous Activity

**Figure 2:**
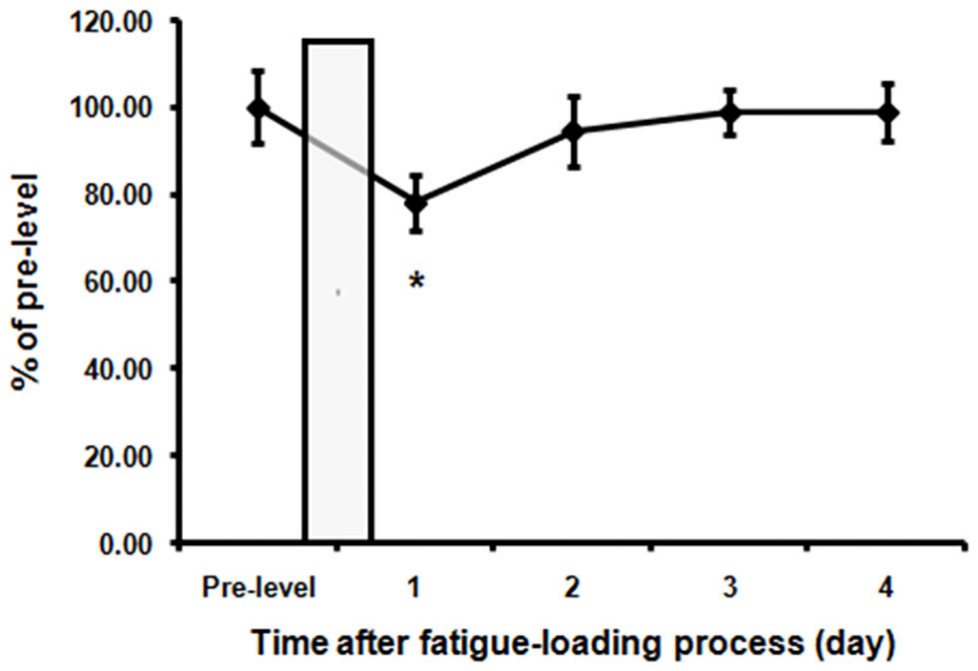
Decreased spontaneous activities in the fatigue group. The nighttime spontaneous activity of the 3 days before fatigue loading was averaged as pre-fatigue baseline. The nighttime spontaneous activity value was normalized to the baseline level. The bar represents the 10-day fatigue loading period. Note that a significant decrease of spontaneous activity in the first night after the end of fatigue loading was observed. Data are expressed as mean ± SEM (*n* = 5), ^*^P < 0.05 vs. pre-fatigue baseline.

Spontaneous activity, known to be associated with motivation which is affected by fatigue, was recorded and analyzed before and after fatigue loading. Figure 2 showed the average activity levels of the rats in the fatigue group during the nighttime before and after fatigue loading. Nighttime spontaneous activity was significantly decreased on the first night after the end of fatigue loading compared to the baseline level (*P* < 0.05), and gradually returned to the baseline level within the following 3 days.

### No Abnormalities Were Observed in Conventional Imaging in Fatigue Group

**Figure 3:**
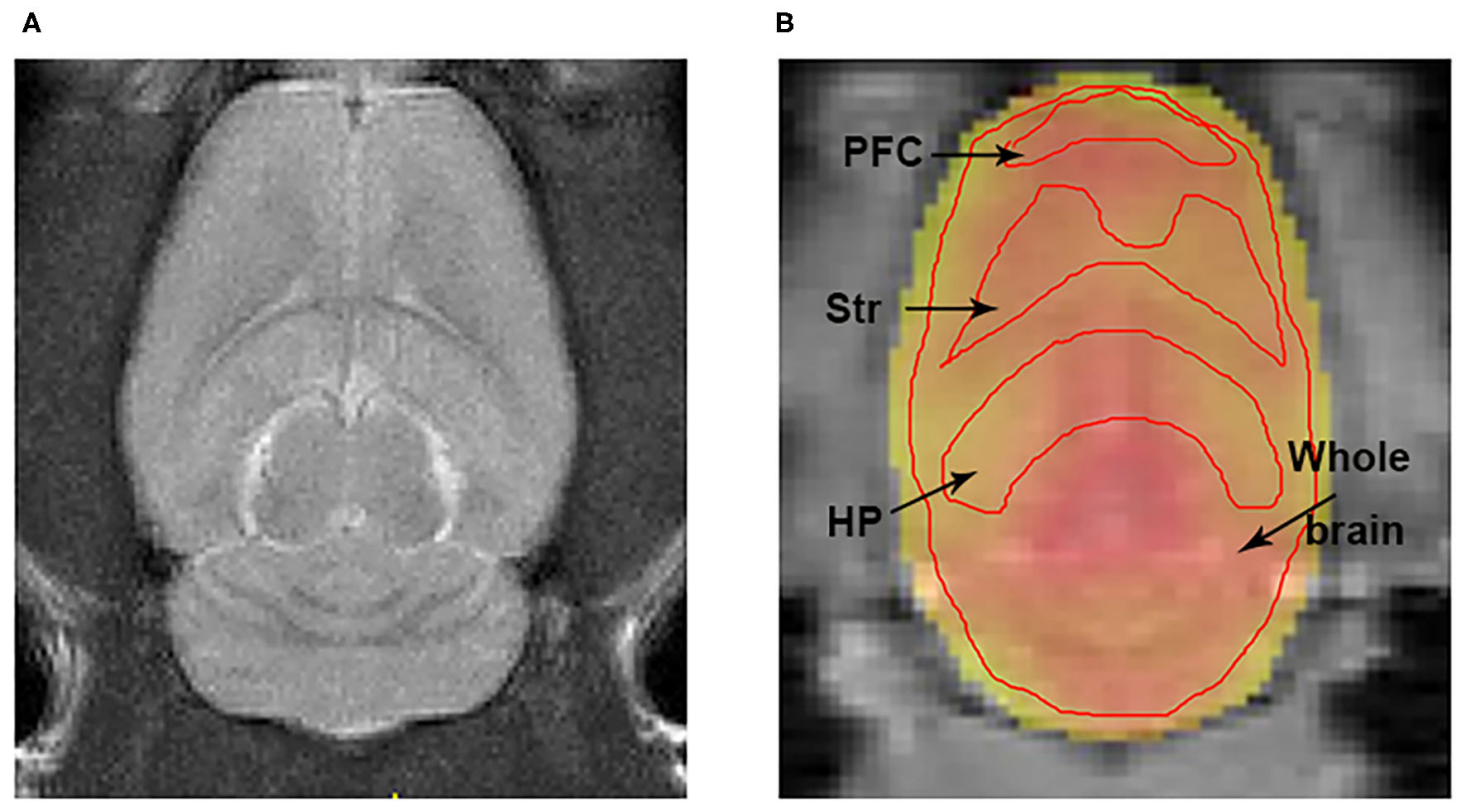
The representations of T2w and Glu-weighted CEST image. **(A)** A representation of T2 weighted image for localization the brain ROIs. **(B)** ROIs for the HP, Str, PFC, and whole brain were manually drawn on the Glu-weighted CEST image. Black arrows indicate ROI tissues. ROI, region of interest; HP, hippocampus; Str, striatum; PFC, prefrontal cortex.

**Figure 4:**
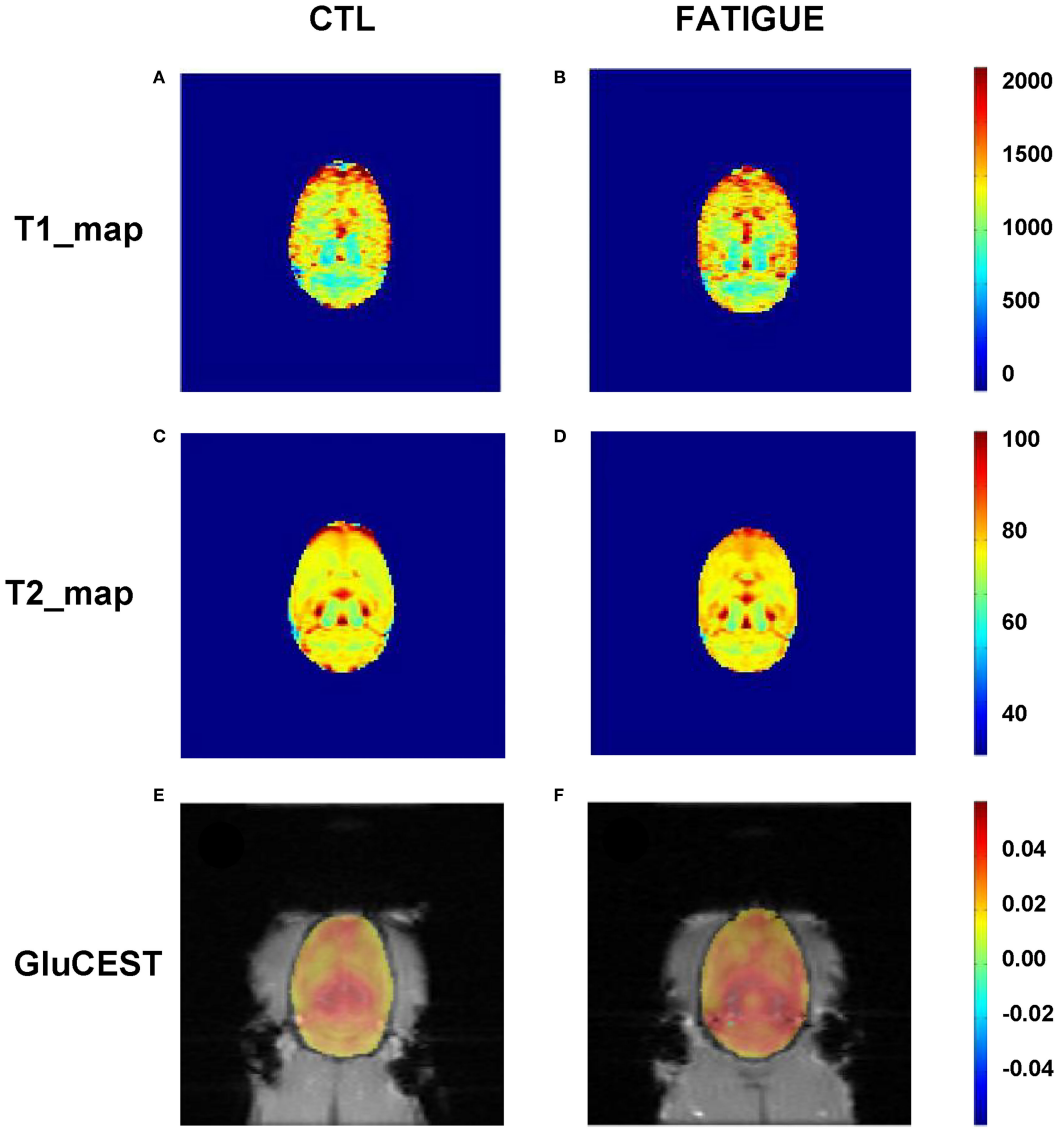
Representative parametric T1 map, T2 map, and Glu-weighted CEST images both in fatigue and control groups. There were no significant differences between the two groups in T1 **(A,B)** and T2 maps **(C,D)**. However, the Glu-weighted CEST images **(E,F)** showed a signal increase in the fatigue group. CTL, control.

The T2-weighted images showed that there were no gross abnormalities in the brain of rats in the fatigue group compared with the control group (Figure 3A). There were no significant differences between the two groups in T1 maps (fatigue group: 1402.7 ± 232.8 ms, control group: 1425.3 ± 269.5 ms) and T2 maps (fatigue group: 75.2 ± 5.7 ms, control group: 74.8 ± 7.5 ms). In contrast, the Glu-weighted CEST images showed a signal increase of about 50% in the whole brain in the fatigue group (Figure 4).

### Glutamate Level Increased in the Whole Brain Measured by Glu-Weighted CEST

**Figure 5:**
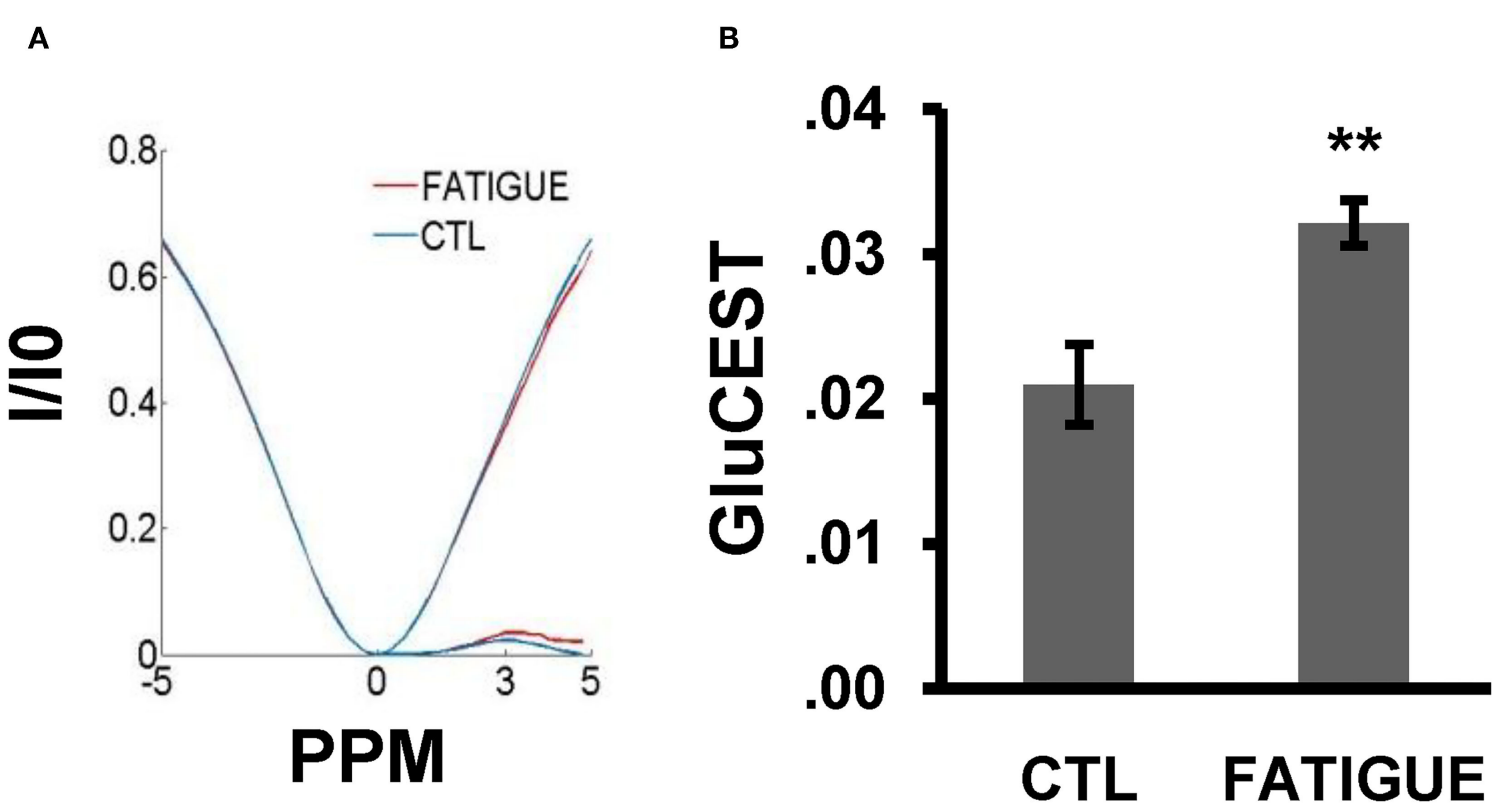
Z-spectral and the glutamate concentration in the whole brain between the two groups. **(A)** In the Z-spectral, a glutamate CEST effect was observed at 3 ppm. Compared to control group, the glutamate CEST effect was increased in the fatigue group. **(B)** The bar graph shows the total glutamate level was significantly higher in the fatigue group than that in the control group. Data are expressed as mean ± SEM (*n* = 10 for the control group, *n* = 6 for the fatigue group), ^**^P < 0.01 vs. control. CTL, control.

In the Z spectrum, a glutamate CEST effect was observed at 3 ppm (Figure 5A). The glutamate CEST measured the glutamate concentration in the whole brain and in different regions of interest (ROIs) in the brain (Figure 3B). We found the glutamate level in the whole brain was significantly higher in the fatigue group compared with the control group (Figure 5B). A further analysis of different sub-regions of brain which were considered relevant to pathophysiology of fatigue including hippocampus, striatum, and prefrontal cortex revealed a trend of increase, although no statistical significance was reached (Table 1).

**Table 1:** Glutamate concentrations measured by Glu-weighted CEST MRI within different regions of brain.

**Brain region**	**CTL**	**FATIGUE**	***P*-value**
WH	0.021 ± 0.003	0.032 ± 0.002	0.004
PFC	0.030 ± 0.002	0.034 ± 0.003	0.302
HP	0.030 ± 0.002	0.034 ± 0.003	0.221
Str	0.023 ± 0.003	0.030 ± 0.003	0.135

*The data were expressed as MEAN ± SEM. WH, whole brain; PFC, prefrontal cortex; HP, hippocampus; Str, striatum; CTL, control*.

## Discussion

In the present study, for the first time Glu-weighted CEST technique was used to measure glutamate content in the brain of rats experiencing the prolonged fatigue loading. It was found that after 10 days of fatigue loading, the glutamate level in the whole brain increased significantly. Further analysis of brain regions implicated in pathophysiology of fatigue, including the prefrontal cortex, hippocampus, and striatum, revealed a trend of increase in the glutamate signals but without reaching a statistical significance.

After 10 days of fatigue loading, the rats showed a significant decrease in nighttime spontaneous activity compared to the pre-fatigue level. This result confirms that our fatigue-loading procedure is sufficient to induce a fatigue state in rats and is consistent with previous reports using this fatigue loading procedure (rest and sleep deprivation) in spite of some modifications in the present study (25, 30). Depressed spontaneous activity is a behavioral manifestation of fatigue, which may reflect an insufficient motivation of fatigued animals to move.

We demonstrate that prolonged fatigue loading induces a significant increase of glutamate level in the whole brain. This result is consistent with most previous reports where acute exhaustive exercise was applied to induce a physical fatigue state in rats (6) or human (4, 5). Acute vigorous exercise causes a globe increase in brain metabolic rate (31, 32) and activates many brain structures (33, 34). The increased glutamate within the CNS during exhaustive exercise is considered to be brain-activity dependent and likely results from an anaplerotic increase in glutamate synthesis via the tricarboxylic acid (TCA) cycle, which may reflect an adaptive shift in brain metabolism when coping with an acute physical fatigue state (4, 6). Metabolic changes and brain activation have also been investigated in rats administrated with complex fatigue-loading procedures like rest and sleep deprivation used in the current study (24, 35). Similar to acute intense exercise, extensive brain activation as shown by c-Fos expression, a marker of neuronal activation, has been observed in fatigued rats after 5-day rest and sleep deprivation (35). This observation indicates that the increased glutamate levels found in the present study may also be, at least partly a result of the activity-dependent increase of glutamate synthesis.

Whereas, the profound metabolic alterations have also been observed in fatigued rats exposed to complex fatigue-loading procedures (rest and sleep deprivation) as used in the current study (24, 36, 37), the changes of metabolic profiles induced by this procedure are not identical to that induced by acute exhaustive exercise. The reduced glucose uptake and insufficient neurotransmitter turnovers were found in fatigued rats induced by deprivation of rest and sleep (24), while the rats experiencing acute vigorous exercise displayed the increased glucose uptake and metabolite turnovers (31). These differences suggest that an impaired, pathological and non-adaptive brain metabolic change may gradually develop after chronic fatigue loading which are different from the physiological, compensatory and adaptive metabolic shift observed in the acute physical fatigue state (4). Supporting this view, a previous study showed that 1 d fatigue loading (rest and sleep deprivation) resulted in increased turnovers of neurotransmitters including dopamine and serotonin, while their turnovers decreased after 5 d fatigue loading, which indicate that the metabolic rates are dynamically regulated along with the prolonged fatigue loading (24).

Increased glutamate levels observed in the present study may also be a result of glial dysfunction induced by chronic fatigue loading. Glial cells, including astrocyte, microglia, and oligodendrocyte, actively participate in the process of glutamate turnover (38). It has been found that rats exposed to 6-day rest and sleep deprivation fatigue loading displayed marked microglia activation around the activated neurons along the spinal cord reflex arc innervating the soleus, an antigravity muscle continuously used to support the body of rat in this fatigue model (39, 40). Moreover, strenuous exercise also disturbed astrocyte function by reducing the expression of GLT-1, one of the EAAT responsible for transferring glutamate into astrocyte, which resulted in an increased extracellular glutamate content (7). These studies indicate that a prolonged fatigue loading may induce glial dysfunction in our fatigue model which could impair glutamate turnover, and consequently, result in an accumulation of glutamate in the brain of fatigued animal. Further study investigating the relationship between glial function and glutamate levels in our fatigue model will be helpful to clarify this issue.

Our result indicates a global increase of glutamate in the whole brain. However, no glutamate changes in specific brain areas, including the prefrontal cortex, hippocampus, and striatum, were observed after chronic fatigue loading. This result suggests that chronic fatigue loading has a general impact on the glutamate levels throughout the whole brain. Although some previous studies showed glutamate alterations in some specific brain areas, such as the hippocampus, cerebellum, after acute intense exercise (6), it is still an unsolved issue which brain areas are implicated in the pathophysiology of fatigue. It has been reported that the glutamate levels in the hypothalamus were higher in MS patients compared to healthy control and correlated with fatigue score (13). However, other imaging studies showed abnormal functional connectivity in the resting state neural network involving the prefrontal cortex, cingulated cortex and insular cortex in CFS patients (41, 42). In contrast, another ^1^H-MRS study find no alteration of combined glutamate and glutamine signals in either occipital cortex or anterior cingulated cortex of CFS patients compared to healthy control or MDD patients (17), and some human studies did not find an association between peripheral glutamate levels with fatigue in CFS patients (14–16). In addition, the dynamic changes of glutamate concentration following fatigue loading have been reported (4, 5, 18). The different outcome among these studies may attribute to different duration and strength of fatigue loading, different species or context. In spite no particular brain area has been consistently identified involving in pathophysiology of fatigue, we speculate that a time, and region dependent glutamate change in CNS may exist and plays an important role in the development of fatigue.

In conclusion, by using Glu-weighted CEST technology, we found a significant increase of glutamate in the whole brain after prolonged fatigue loading in a complex fatigue-inducing animal model. Our result indicates that glutamate metabolism may play important roles in the pathophysiology of fatigue.

## Data Availability Statement

The datasets presented in this study can be found in online repositories. The name of the repositories is Open Science Framework. The data can be found at: https://osf.io/js8au/.

## Ethics Statement

The animal study was reviewed and approved by the Ethics Committee of Shantou University Medical College on Animal Care and Use.

## Author Contributions

RL, ZD, HX, QH, YC, and HZh contributed to conception and design of the study. RL, ZD, HZe, and ZZ performed Glu-weighted CEST and MRI imaging. RL, DH, and ZF conducted behavioral tests. RL, ZD, and HZh performed the statistical analysis. RL, ZD, DH, and HZh wrote sections of the manuscript. All authors contributed to manuscript revision, read, and approved the submitted version.

## Conflict of Interest

The authors declare that the research was conducted in the absence of any commercial or financial relationships that could be construed as a potential conflict of interest.
